# Titania: an integrated tool for in silico molecular property prediction and NAM-based modeling

**DOI:** 10.1007/s11030-025-11196-5

**Published:** 2025-04-23

**Authors:** Nikoletta-Maria Koutroumpa, Maria Antoniou, Dimitra-Danai Varsou, Konstantinos D. Papavasileiou, Nikolaos K. Sidiropoulos, Christoforos Kyprianou, Andreas Tsoumanis, Haralambos Sarimveis, Iseult Lynch, Georgia Melagraki, Antreas Afantitis

**Affiliations:** 1 NovaMechanics Ltd, 1070 Nicosia, Cyprus; 2 School of Chemical Engineering, National Technical University of Athens, 157 80 Athens, Greece; 3 Entelos Institute, 6059 Larnaca, Cyprus; 4 Computation-Based Science and Technology Research Center, The Cyprus Institute, 2121 Nicosia, Cyprus; 5 NovaMechanics MIKE, 18545 Piraeus, Greece; 6 School of Geography, Earth and Environmental Sciences, University of Birmingham, Birmingham, UK; 7 Division of Physical Sciences & Applications, Hellenic Military Academy, 166 73 Vari, Greece

**Keywords:** Quantitative structure–property/toxicity relationships, Machine learning, Property prediction, Enalos cloud platform, Isalos analytics platform, Titania web tool

## Abstract

Advances in drug discovery and material design rely heavily on in silico analysis of extensive compound datasets and accurate assessment of their properties and activities through computational methods. Efficient and reliable prediction of molecular properties is crucial for rational compound design in the chemical industry. To address this need, we have developed predictive models for nine key properties, including the octanol/water partition coefficient, water solubility, experimental hydration free energy in water, vapor pressure, boiling point, cytotoxicity, mutagenicity, blood–brain barrier permeability, and bioconcentration factor. These models have demonstrated high predictive accuracy and have undergone thorough validation in accordance with OECD test guidelines. The models are seamlessly integrated into the Enalos Cloud Platform through Titania (https://enaloscloud.novamechanics.com/EnalosWebApps/titania/), a comprehensive web-based application designed to democratize access to advanced computational tools. Titania features an intuitive, user-friendly interface, allowing researchers, regardless of computational expertise, to easily employ models for property prediction of novel compounds. The platform enables informed decision-making and supports innovation in drug discovery and material design. We aspire for this tool to become a valuable resource for the scientific community, enhancing both the efficiency and accuracy of property and toxicity predictions.

**Supplementary Information:**

The online version contains supplementary material available at 10.1007/s11030-025-11196-5.

## Introduction

Accurate and reliable prediction of molecular properties is essential in the drug discovery process and for rational material design. In silico methods comprise a range of computational strategies for studying the properties and behavior of chemical compounds. These methods include quantitative structure–activity relationships (QSAR) and quantitative structure–property relationships (QSPR). QSAR/QSPR models aim to establish correlations between endpoints—such as biological activity against a target or physicochemical properties—and features of chemical compounds encoded in their molecular structure. Such models can be later applied to predict the properties of untested chemicals [1]. Similar computational approaches, quantitative structure–toxicity relationships (QSTR), can assess the toxicity of chemicals. Therefore, QSAR-type models enable the prediction of chemical endpoints, even prior to the actual synthesis of the molecules/materials of interest, using only their molecular structure information [2]. Minimizing toxicity and optimizing specific properties are critical for designing safe medicines or advanced materials, where incorrect estimation of these parameters may cause undesired side effects [3, 4].

Currently, computational chemistry is entering the age of big data, where a vast amount of data suitable for QSAR modeling is available. However, data quality has become one of the fundamental parameters to consider when compiling datasets. In 2004, the Organization for Economic Cooperation and Development (OECD) developed a set of guidelines for researchers to achieve the regulatory acceptance of QSAR models [5]. These guidelines include the definition of endpoints, the use of unambiguous algorithms, definition of the domain of applicability of the models, the ability to measure the goodness-of-fit, robustness, and predictivity of the models, and—if possible—the mechanistic interpretation of the models. Following these guidelines, validated QSAR-type models can be developed, which can prioritize compounds for experimental evaluation, by excluding from further steps those compounds that do not meet the criteria of interest. To ensure that the developed QSAR models are properly documented, reliable, shareable, and suitable for making informed decisions, the European Commission’s Joint Research Centre (JRC) developed the QSAR Model Reporting Format (QMRF) documentation standard. This standardized template is used for reporting the key information on QSAR models, ensuring that they are transparently documented and that their validity can be assessed [6].

Machine learning (ML) algorithms, and more recently its subset deep learning (DL), have been applied to derive statistical QSAR-type models. Chemical structures can be related to changes in biological activity, physicochemical properties, or toxicity. Compound structures are often numerically encoded using predefined features (descriptors) or molecular fingerprints, and ML models learn a mapping of molecular features to endpoints. Even though correlations between features and endpoints do not imply causality, ML models are able to predict properties for new substances. Some models have the ability to extrapolate from the training data, enabling prospective use to predict properties of new chemical series [7]. Nevertheless, ML models may suffer from the “missing fragment problem”, when new chemical fragments are encountered that were not present in the training set. This issue can be addressed by employing a larger training dataset and considering the applicability domain of the model [8].

QSAR is today a mature field, with several successful applications of ML for the identification of novel compounds with improved properties [9–11]. Beyond physicochemical properties and biological activity, QSAR/QSPR models are also widely used for predicting ADMET (Absorption, Distribution, Metabolism, Excretion, and Toxicity) properties, which are critical for assessing drug safety and efficacy [12]. Accurate ADMET modeling helps prioritize compounds with favorable pharmacokinetic and toxicity profiles, reducing late-stage failures in drug development [13–15]. QSAR models are also a key component of New Approach Methodologies (NAMs), supporting the move toward more ethical, efficient, and mechanistically informed methods of assessing chemical safety. Additionally, it is observed that ultimately models do not remain within the developers’ group but are disseminated to the broader stakeholder community to serve as an important tool in future novel compound design. Several studies in the literature describe the development of user-friendly web applications to support the uptake and wider re-use of predictive models [16, 17]. At the same time, various databases and cloud platforms have emerged to host QSAR models (e.g., the QsarDB [18], the molinspiration [19] etc.) and consequently make these models more Findable, Accessible, Interoperable, and Reusable (FAIR).

Numerous QSAR modeling tools have been developed to automate predictive modeling, but they often come with limitations that hinder usability, accessibility, or flexibility. AutoQSAR is a machine learning application for automated QSAR modeling [20]. Similarly, eTOXlab offers an alternative automated QSAR framework [21]. Both require significant technical expertise, a deep understanding of molecular modeling and python programming skills to be used by the research community. Cox et al. [22] developed a Pipeline Pilot web application (QSAR Workbench), which is only restricted to Pipeline Pilot uses. SwissADME [23] and admetSAR [24] are web tools that give free access to a pool of predictive models for physicochemical properties, pharmacokinetics, and medicinal chemistry friendliness. ADMETlab 2.0 and ADMETlab 3.0 significantly expand upon its predecessor, with an increase in supporting endpoints and an incorporation of graph attention framework for improved accuracy across several ADMET-related endpoints [25, 26]. The aforementioned web tools are well established and widely used for molecular property predictions.

In this work, we first aim to develop robust and accurate QSPR/QSTR models for nine properties of small molecules: octanol/water partition coefficient, water solubility, experimental hydration free energy in water, vapor pressure, boiling point, cytotoxicity, mutagenicity, blood–brain barrier permeability, and bioconcentration factor. The models are developed, documented, and validated according to the OECD guidelines, ensuring transparency, reliability, and regulatory compliance. Unlike several existing QSAR web tools, our platform integrates a 3D visualization field to inspect the molecule of interest, an applicability domain check to assess the reliability of each prediction and the training compounds that were used in each prediction, enhancing interpretability and trust in the results. Additionally, our models are released through the Enalos Cloud Platform, via the Titania web suite, offering a user-friendly interface that is accessible to both experts and non-experts. Finally, all of the datasets, including the compounds’ descriptors and properties/toxicity, used to develop the QSPR/QSTR models are made available through the ChemPharos database [27], a specialized database designed for advanced chemical data management, and are ready-for-modeling by any interested user, further promoting reproducibility and advancement of QSAR research.

## Materials and methods

### Datasets

For the development of the predictive models, we collected nine datasets that had been previously used for building QSPR/QSTR models. The assessed endpoints were the following: octanol/water partition coefficient (P), water solubility (S), experimental hydration free energy in water (Free-Solv), vapor pressure (VP), boiling point (BP), cytotoxicity, mutagenicity, blood–brain barrier permeability (BBB), and bioconcentration factor (BCF).

Table 1 shows the total number of compounds used for modeling and validation of the predictive models. The initial datasets were split into training and test (validation and blind) sets either randomly, or using a representative sampling method, such as Kennard and Stone [28, 29]. The specific splitting method used for each model is detailed in the corresponding QMRF and the subset (training, validation or blind) to which the compounds belong is clearly indicated within the ChemPharos datasets.

**Table 1:** Number of compounds included in the datasets for the development and validation of the QSPR/QSTR models

Property	Total	Train	Validation	Blind
logP	14,207	10,655	2842	710
logS	2010	1508	402	100
Free-Solv	642	481	129	32
logVP	2713	2034	544	135
BP	5432	4074	1087	271
Cytotoxicity	5416	3791	1462	163
Mutagenicity	5738	4303	1148	287
BBB	7807	5855	1562	390
logBCF	608	456	122	30

The octanol/water partition coefficient (P) is typically expressed in logarithmic form (logP) and quantifies a compound’s tendency to partition between octanol and water, with logP values reflecting its lipophilicity and influencing drug bioavailability [30, 31]. A compound’s aqueous solubility (S), typically represented as logS, indicates the quantity of the substance that can dissolve in water [32]. The Free-Solv model was trained on 642 molecules from MoleculeNet containing the experimental free energy of these molecules in water [33]. Vapor pressure (VP), often denoted as logVP, is a physical property that describes a substance’s tendency to evaporate or condense. Boiling point (BP), on the other hand, serves as an indicator of a substance’s volatility. Cytotoxicity, describing the toxic effects on NIH/3T3 fibroblasts, was modeled using 5416 compounds from PubChem’s BioAssay 651744 [34], which classified chemicals as cytotoxic or non-cytotoxic based on activity thresholds. Mutagenicity, assessed via the Ames test, evaluates a compound’s potential to cause genetic mutations; our model leveraged 5738 substances retrieved from the EPA EPI Suite [35, 36] which was based on the benchmark dataset of Hansen et al. [37]. Predicting permeability through the BBB, which serves as a primary defense mechanism, shielding the brain from exposure to potentially toxic substances, is a challenging task in drug development. We trained a predictive model using a curated database of BBB permeability data containing 7807 compounds with their permeability class (permeable/non-permeable) [38]. Finally, bioconcentration, measured by the BCF (logBCF), reflects a chemical’s potential to accumulate in organisms, posing ecological risks [39]. The data to model logP, logS, logVP, BP, and logBCF were retrieved from the study of Zang et al. [8].

All the selected datasets were curated on the original study from where they were retrieved. More specifically, data retrieved from Zang et al. [8] were curated based on workflows developed at EPA to provide QSAR-ready datasets free from structural ambiguities. Similarly, the Mutagenicity dataset was curated from EPA EPI Suite [35, 36] and Free-Solv was already curated on MoleculeNet [33]. BBB permeability data were retrieved from Meng et al. [38], where compounds were curated using ChEMBL standardization and neutralization procedures. Data curation is a crucial step in the development of QSPR/QSTR models [40, 41]. For that reason, in addition to the curation performed in the original studies, we conducted a further curation process on all datasets. This involved checking chemical structures, identifying and removing duplicate entries, and addressing any missing values to ensure consistency across the data.

The results of the predictive models for the nine properties will be presented based on the type of endpoints of the properties, meaning regression and classification endpoints. Tables 2 and 3 show the statistics of each dataset for regression and classification properties. More information regarding the datasets is available in the QMRF of each model which is included in the Supplementary Material.

**Table 2:** Statistics of regression datasets

Property	Min	Max	Mean ± std
logP	− 5.40	11.29	2.07 ± 1.83
logS	− 12.06	1.58	− 2.60 ± 2.19
Free-Solv	− 25.47	3.43	− 3.80 ± 3.94
logVP	− 13.68	5.67	− 2.04 ± 3.57
BP	− 88.60	548.00	188.98 ± 85.07
logBCF	− 0.35	5.97	1.88 ± 1.26

**Table 3:** Total compounds in each class for classification datasets

Property	Class 0	Class 1	Ratio Class 1/Class 0
Cytotoxicity	2307	3109	1.35
Mutagenicity	2594	3144	1.21
BBB	2851	4956	1.74

### Molecular features

Featurization of the compounds, i.e., conversion of varied forms of data into numerical data that can be used as input to ML algorithms, was performed using the Mold2 software package, developed by the Center for Toxicological Research (NCTR) [42] for the retrieval of molecular descriptors encoding the characteristics of small molecules. Requiring solely the SMILES notations in SDfile format as input, Mold2 calculates 777 different one-dimensional and two-dimensional descriptors based on the structure of each compound. One-dimensional descriptors are related to the number of specific atoms, whereas the two-dimensional descriptors mainly refer to bond and functional groups information, physicochemical properties, and structural features, such as charge, connectivity, and topological and autocorrelation indices [42]. Mold2 is available as an extension in KNIME through the Enalos + nodes [43]. The datasets including the calculated descriptors are disseminated through the ChemPharos database [27] in a tabular/ready-for-modeling format to support cheminformatics analysis by any interested research group.

### Data pre-processing

The first step of data pre-processing involved removing columns with redundant data, based on the existence of repetitive values [43, 44]. Additionally, the raw data were pre-processed with a Low Variance filter to minimize the dimensionality of the dataset and exclude descriptors that have a minor impact on the target variable. This process involved setting a threshold limit of 20% for the following properties: logP, logS, Free-Solv, logVP, BP, logBCF, Mutagenicity, and BBB. For Cytotoxicity, a threshold limit of 10% was applied. Descriptors whose variance fell below these thresholds were excluded from further analysis. The low variance filtering was performed before the division of the dataset into training, validation, and blind sets, using the relevant node “Low Variance Filter” on KNIME.

For normalization, the z-score normalization method (Gaussian distribution) was used, and the values of descriptors were transformed to have a mean value of zero and a standard deviation of one. Endpoint values, meaning the property values, were excluded from this step. For datasets with continuous endpoints, values were represented in a logarithmic format, except for the BP dataset which contains negative values and Free-Solv, in which the range of experimental values is relatively small making the logarithmic transformation unnecessary. Logarithmic-transformed values reduce the wide range of magnitudes and make interpretation of the results easier. Normalization was carried out only on the training dataset and then applied to the validation and blind datasets.

An important part of data pre-processing is feature selection [45]. In our case, a large number of descriptors were computed for each molecule. The feature selection process was carried out on the training datasets, and later the validation and blind datasets were filtered based on the selected subsets of features. To select the descriptors that are highly correlated with the target property, we used the BestFirst variable selection method and the CfsSubsetEval (CFS) evaluator. CFS subset evaluator identifies a subset of uncorrelated variables considering their individual ability to predict the endpoint and then searches for the possible combinations of variables and selects the best one using the BestFirst search method, which performs greedy hill climbing with backtracking. In the mutagenicity classification model, the InfoGain variable selection with Ranker evaluator was used [46]. InfoGain measures the information gain of an attribute with respect to the class (endpoint), whereas Ranker ranks attributes and removes the lower-ranking ones [46]. For the pre-processing steps, we used KNIME to apply these variable selection methods and perform descriptor filtering.

### Model development

As noted in the literature, there is no single optimal ML algorithm for all potential data modeling problems [47, 48]. However, one can examine several algorithms and select the one that best correlates the input data with the endpoint. In our case, we examined k-nearest neighbors (kNN), random forest (RF), support vector machines (SVM), and multi-layer perceptron (MLP) for both regression and classification tasks. The kNN and RF methodologies were found to be the most appropriate based on their performance on the validation set. kNN is an instance-based learning technique that classifies an instance according to the weighted majority vote of the k closest training examples (neighbors), in classification problems. In regression, the weighted average of the response variable for the identified neighbors is used to generate the prediction. The inverse distance values serve as the weighting factors in both cases. The kNN functionality not only provides a prediction of the response variable, but also offers the possibility to provide for each query compound the k neighbors and their Euclidean distances [44]. Therefore, it is possible to visualize the entire predictive space of each study and use it within a read-across framework [49].

The RF methodology was chosen for the discrimination of compounds based on their mutagenicity. The RF model is an ensemble tree-based learning algorithm; that is, the algorithm averages predictions over many individual decision trees. It combines two concepts, the Bagging technique and the Random Selection of Features [50]. A set of T decision trees is generated based on bootstrap sampling from the original training data. For each node, the optimal splitting feature is selected from a set of n features that are randomly picked from a total of N features. Random forests have gained popularity due to their high accuracy predictions even with limited samples and a large number of features, due to the embedded feature selection in the model generation process [50–52].

### Evaluating ML models

In order to ensure the obtained QSPR/QSTR models have good generalization ability for new chemical entities, an external validation scheme was applied. In this approach, the initial dataset was divided into training, validation, and blind subsets. The training set was used to determine the optimal subset of descriptors for the endpoint prediction and to define the modeling parameters. These parameters were tuned by assessing the performance of the developed model using the validation set until optimized modeling parameters were achieved. Furthermore, the validation set was used for model selection. The final predictive accuracy was assessed using the blind set, which did not take part in the model development procedure.

Data splitting can be performed either randomly or using a deterministic method such as the Kennard and Stone partitioning algorithm [28, 29]. This algorithm is commonly used in QSAR studies as it selects representative subsets of the descriptor space, giving more confidence in future predictions of test set molecules that come from the same distribution of the training set [53]. To ensure the robustness of the developed models, we performed internal validation tests, such as leave-k-out cross-validation. For the training set produced by the initial splitting of data, we performed leave-one-out (LOO) cross-validation and fivefold cross-validation. To verify the reliability and predictive ability of the models, we also performed Y-randomization tests. In the Y-randomization test, the vectors containing the endpoints are randomly shuffled and are used to develop new predictive models. If the models present lower validation metrics compared to the original model, then the possibility of chance correlation on the original model is minimized.

Model validation was also accompanied by several statistical measures to describe and assess a model’s performance. We applied different statistical metrics for regression and classification methods. The regression metrics were the correlation of determination (R^2^), cross validated R^2^ (Q^2^), external explained variance (Q_ext_^2^), mean absolute error (MAE), root mean squared error (RMSE), and concordance correlation coefficient (CCC). The classification metrics were sensitivity (SEN), specificity (SPE), accuracy (ACC), and Matthew’s correlation coefficient (MCC). More details regarding the evaluation metrics are available in the Supplementary Material.

### Applicability domain

Definition of the domain of applicability (DoA) is necessary for describing the limitations of a model since it determines the area of input data for which the model is expected to make accurate (interpolated) predictions. Compounds that fall outside the model’s applicability domain limits are filtered out, as the model would provide unreliable results (extrapolated predictions) [54]. Importantly, the size of the DoA is fully defined by the size and diversity of the modeling dataset and the computational method used in the development of the QSPR/QSTR model.

Different approaches can be used to specify the degree of similarity between a compound of interest and the compounds included in the training set. All the models described in this work employ a distance-based method to define the DoA. This approach relies on the use of the Euclidean distances between existing data points of the training set to estimate whether a model’s assumptions are valid for new observations. Specifically, a predefined fixed boundary, the DoA threshold, is calculated as

1 $$threshold=d+Z\sigma$$

where *d* is the average of the distances included in the subset of distances that are lower than the mean value of all distances between all training samples, *σ* is the standard deviation of all distances included in the subset of distances that are lower than the mean of all distances, and *Z* is an empirical parameter, with a default value of 0.5. The distance of an unknown compound to its nearest neighbor in the training set is compared to the threshold, and if the value exceeds the DoA threshold, then the prediction is considered unreliable.

### Web server implementation

The Enalos Cloud Platform (https://www.enaloscloud.novamechanics.com) is a comprehensive suite of predictive models and tools offered as ready-to-use web services across cheminformatics, nanoinformatics, and materials engineering [55, 56]. Developed using the ZK framework (an open-source Ajax web application framework), JavaScript, and Java, the platform integrates robust open-source libraries and software, including Python, R, LAMMPS, OpenKIM, NGL Viewer, MD Analysis, KNIME, WEKA, ImageJ, and ParaView, along with proprietary tools, such as Enalos + nodes. These web services do not require programming expertise, which means that any user can generate reliable predictions to assess the toxicity of small molecules and materials. Conceived to broaden access, through a free-to-use and intuitive interface, the platform facilitates access to advanced computational tools to reduce extensive experimental work and favor alternatives to animal testing, thus further promoting informed decision-making in scientific research and innovation. In this course, the models developed within this work are delivered through an easy-to-use web-service called Titania hosted on Enalos Cloud. Titania allows the simultaneous assessment of multiple endpoints of small molecules with one request, allowing in this way virtual screening of large sets of compounds and contributing to drug and material design projects.

Furthermore, simple molecular descriptors and properties are compiled in the web interface. The number of hydrogen bond donors, number of hydrogen bond acceptors, number of rings, and number of rotatable bonds are calculated using the Lipinski module of RDKit, and molecular weight, formula, and topological polar surface area (TPSA) are also calculated by RDKit [57]. Four more medicinal chemistry properties are available, the drug-likeness (QED), which quantifies the drug-likeness of a molecule, ranging in [0,1] [58, 59], the Synthetic Accessibility score (SAscore), providing an estimation of the synthetic accessibility of a molecule, transformed in range [0,1] [60], one indication whether the compound of interest has properties consistent with the Lipinski’s Rule of five (Ro5) and whether it can be found in PAINS (Pan-Assay Interference compounds) alerts.

## Results and discussion

The goal of this study was to develop predictive models for the in silico assessment of a set of physicochemical properties and toxicity endpoints of small molecules using computationally derived descriptors encoding their structural properties. The KNIME (Konstanz Information Miner) Analytics Platform was used to perform compounds’ featurization, by employing the Enalos + Mold2 node and for model development [43]. The Enalos + Main PubChem node was also employed in order to extract information for the query compounds from the PubChem database and enrich the datasets uploaded in ChemPharos with information on their PubChem Compound IDs, InChI, Molecular formula, etc. whenever available.

**Fig. 1:**
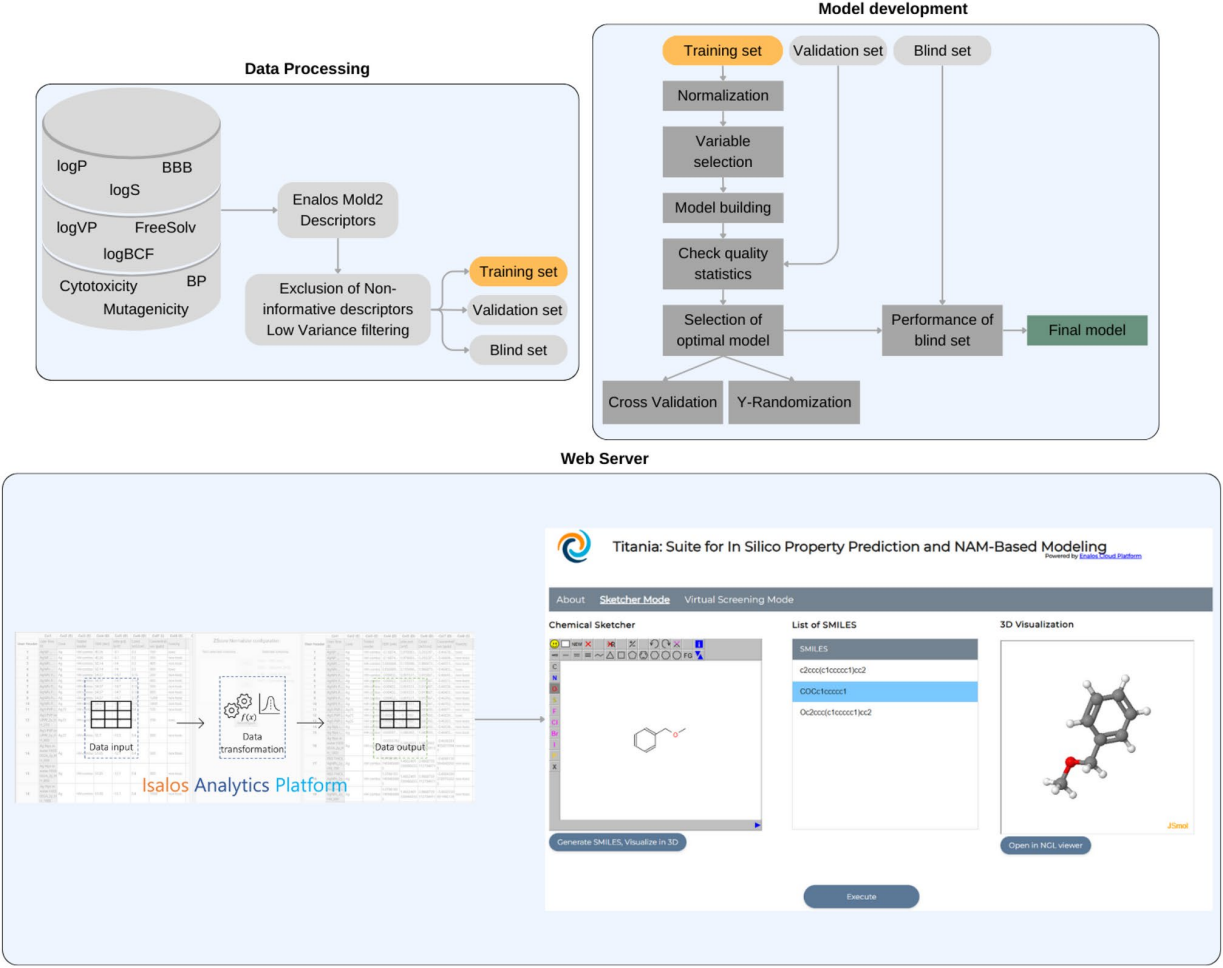
Schematic representation of the development and use of the Titania models. Molecular featurization, data pre-processing, and model development were performed on KNIME, and models were transferred into the Isalos Analytics Platform. The developed models were easily deployed through the Enalos Cloud Platform as a user-friendly web-service called Titania

All nine datasets with different input variables and endpoints were handled with the same pre-processing and modeling workflow in KNIME and were later transferred into the Isalos Analytics platform, which is an intuitive tool that allows data manipulation and robust model development by non-programmers, to facilitate web-implementation [44]. The workflow consists of importing the calculated descriptors, pre-processing, splitting of data, variable selection, modeling, validation of the trained model, and DoA determination steps (Fig. 1). Isalos permits deployment and sharing of the models as web services for straightforward access by the broader community, via the Enalos Cloud Platform. The developed models are made available through a user-friendly web-service in the Enalos Cloud, i.e., Titania web tool (https://enaloscloud.novamechanics.com/EnalosWebApps/titania/).

The key results of the LOO cross-validation are presented in tabular format in Tables 4 and 5 and in detail in the reports adhering to OECD QMRF guidelines [61], available as supporting information files accompanying this work.

**Table 4:** Performance of the regression models for LOO cross-validation

Property	Q^2^	MAE	RMSE	CCC
logP	0.808	0.577	0.810	0.892
logS	0.835	0.649	0.871	0.910
Free-Solv	0.809	1.019	1.699	0.895
logVP	0.894	0.757	1.160	0.943
BP	0.867	21.28	31.049	0.928
logBCF	0.687	0.558	0.728	0.822

**Table 5:** Performance of the classification models for LOO cross-validation

Property	SEN	SPE	ACC	MCC
Cytotoxicity	0.852	0.731	0.801	0.590
Mutagenicity	0.799	0.729	0.767	0.529
BBB	0.931	0.766	0.871	0.717

### Models

To determine the optimal ML algorithm for each property, we evaluated kNN, RF, SVM, and MLP. For each property, we used the training sets for variable selection, the validation sets for hyperparameter tuning, and model selection between the different ML algorithms and the blind sets for the assessment of the final model. The performance of these algorithms on validation subsets is available in Supplementary Material Tables S1 and S2 . Model selection was based on key performance metrics, such as R^2^, MAE, RMSE, CCC for regression models, and sensitivity, specificity, accuracy, and MCC for classification models. While RF was selected for mutagenicity due to its robust performance, for all other endpoints, kNN and RF performed similarly. kNN was chosen due to its similar predictive performance, faster computational efficiency, and interpretability. In particular, the kNN model offers the possibility to provide for each query compound the nearest neighbors and their Euclidean distances. Therefore, it is possible to visualize the predictive space and use it within a read-across framework. This interpretability feature is an important advantage in practical applications where understanding the rationale behind predictions is critical.

To ensure the robustness and predictive reliability of our models, we employed a comprehensive validation framework, including LOO cross-validation, fivefold cross-validation, Y-randomization, and domain of applicability assessment. The performance of six regression models developed for logP, logS, Free-Solv, logVP, BP, and logBCF is presented in Table 4 (LOO cross-validation) and Table 6 (comparison of training, validation and blind dataset). With the LOO cross-validation, the Q^2^ achieved values ranging from 0.69 to 0.89 for all six models. The externally explained variance of the blind dataset, Q_ext_^2^, reached values of 0.804 for logP, 0.922 for logS, 0.859 for Free-Solv, 0.882 for logVP, 0.811 for BP, and 0.895 for logBCF, demonstrating generalizability across multiple endpoints. The other evaluation metrics for training, validation, and blind subsets are shown in Table 6.

**Table 6:** Performance of regression models for training, validation, and blind datasets

Property	Dataset	R^2^	MAE	RMSE	CCC
logP	Train	0.999	0.050	0.071	0.999
	Validation	0.850	0.487	0.682	0.914
	Blind	0.794	0.512	0.763	0.877
logS	Train	1.000	0.023	0.030	0.999
	Validation	0.870	0.524	0.699	0.922
	Blind	0.849	0.541	0.682	0.916
Free-Solv	Train	0.999	0.060	0.150	0.999
	Validation	0.856	0.879	1.436	0.922
	Blind	0.884	0.667	1.153	0.935
logVP	Train	1.000	0.037	0.052	0.999
	Validation	0.893	0.736	1.148	0.943
	Blind	0.881	0.817	1.338	0.934
BP	Train	1.000	0.756	1.469	0.999
	Validation	0.869	20.174	30.872	0.929
	Blind	0.833	23.874	33.842	0.911
logBCF	Train	0.998	0.030	0.055	0.999
	Validation	0.833	0.350	0.445	0.911
	Blind	0.897	0.302	0.378	0.942

Tables 5 and 7 summarize the performance of three classification models developed for cytotoxicity, mutagenicity, and BBB. Table 5 presents the results for LOO cross-validation, where prediction accuracies range from 77 to 87%. Sensitivity, specificity, and Matthew’s correlation coefficient (MCC) further highlight the balance between true positives and negatives, providing a comprehensive view of classification accuracy and reliability. Table 7 details model performance across the training, validation, and blind sets. The predicted accuracies for cytotoxicity, mutagenicity, and BBB for the external blind dataset were 0.85, 0.79, and 0.86, respectively.

**Table 7:** Performance of classification models for training, validation, and blind datasets

Property	Dataset	SEN	SPE	ACC	MCC
Cytotoxicity	Train	0.943	0.873	0.913	0.822
	Validation	0.857	0.770	0.820	0.630
	Blind	0.894	0.797	0.853	0.697
Mutagenicity	Train	0.994	0.991	0.992	0.984
	Validation	0.792	0.811	0.802	0.602
	Blind	0.772	0.806	0.787	0.576
BBB	Train	0.985	0.980	0.983	0.963
	Validation	0.940	0.753	0.872	0.720
	Blind	0.933	0.737	0.864	0.696

We observed that while our models demonstrated strong performance on the training set, their performance on validation and blind sets decreased, which is anticipated due to the bias-variance tradeoff [62]. A balanced model aims to minimize both variance and bias to achieve optimal generalization. However, even with a balanced model, a small drop in accuracy from training to test data is expected since the model is exposed to unseen data. In our case, models’ performance on unseen data remains consistent with general acceptability criteria for all models, with R^2^ ranging from 0.794 to 0.897 on regression models and accuracies ranging from 0.787 to 0.864 on classification models [41, 63].

To further assess models’ generalizability, Y-randomization tests were performed, where randomized datasets resulted in significantly lower predictive performance, confirming that our models are learning meaningful structure–activity relationships rather than random noise. Additionally, the domain of applicability was defined, ensuring that predictions were made within a well-characterized chemical space, confirming that models are consistent with QSAR acceptability criteria. The results of fivefold cross-validation provide a more robust estimate of generalization error, confirming the overall stability of our models. While models meet QSAR acceptability criteria, further refinement through feature selection, data augmentation, or regularization strategies could enhance generalizability of the models in future work. Details of the model development, including fivefold cross-validation and Y-randomization results, are available in the QMRF reports presented (one for each model) in the Supplementary Material.

### Descriptor contributions and model overlap

**Fig. 2:**
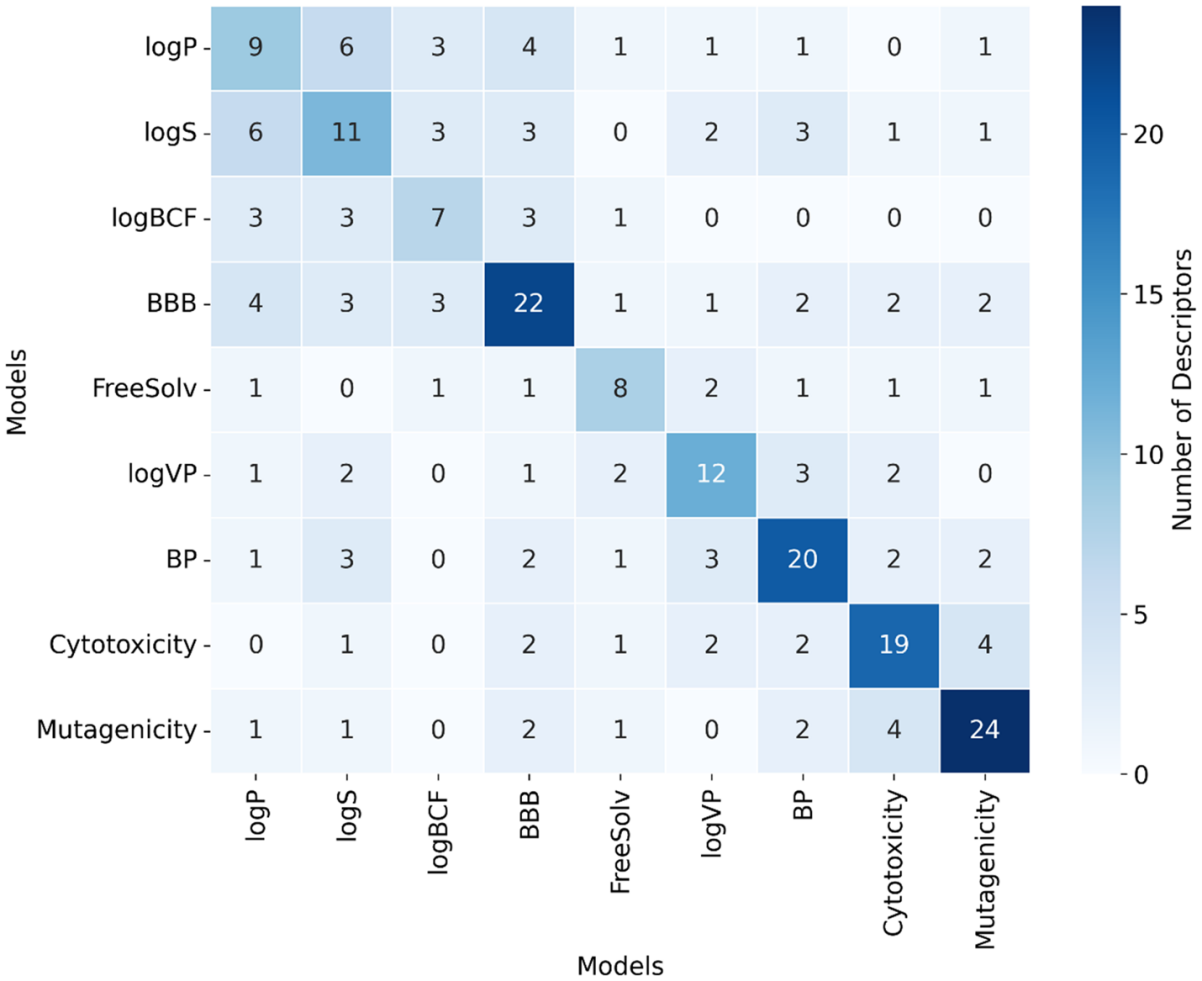
Distribution of Mold2 descriptors across the 9 predictive models. The diagonal line indicates the number of descriptors needed per model, while the rows and columns indicate the number of descriptors shared by each pair of models

Molecular descriptors play a crucial role in developing predictive models by capturing key physicochemical and structural properties of compounds. Understanding how descriptors contribute to different models and identifying overlaps in predictive descriptors between models can provide insight into shared underlying mechanisms among properties. In this study, Mold2 descriptors were selected to represent the chemical structures. As shown in Fig. 2, logP and logS have a strong overlap of descriptors, sharing six out of the nine descriptors needed for prediction of logP. This overlap highlights the correlation between these models, as both are influenced by molecular polarity and hydrophobicity of the compounds. Similarly, logP and BBB share 4 descriptors with BBB prediction needing 22 descriptors in total as shown in Fig. 2, highlighting the correlation between compound permeability and lipophilicity [64]. Free-Solv, representing free solvation energy, exhibits minimal overlap with most models and 8 descriptors are needed to model this property (Fig. 2). This suggests that the descriptors for solvation properties of compounds are distinct from those driving toxicity or partition coefficient. Cytotoxicity and Mutagenicity share four descriptors, reflecting their reliance on similar chemical features probably associated with biological effects, such as membrane/receptor binding.

### Implementation of the Titania web server

**Fig. 3:**
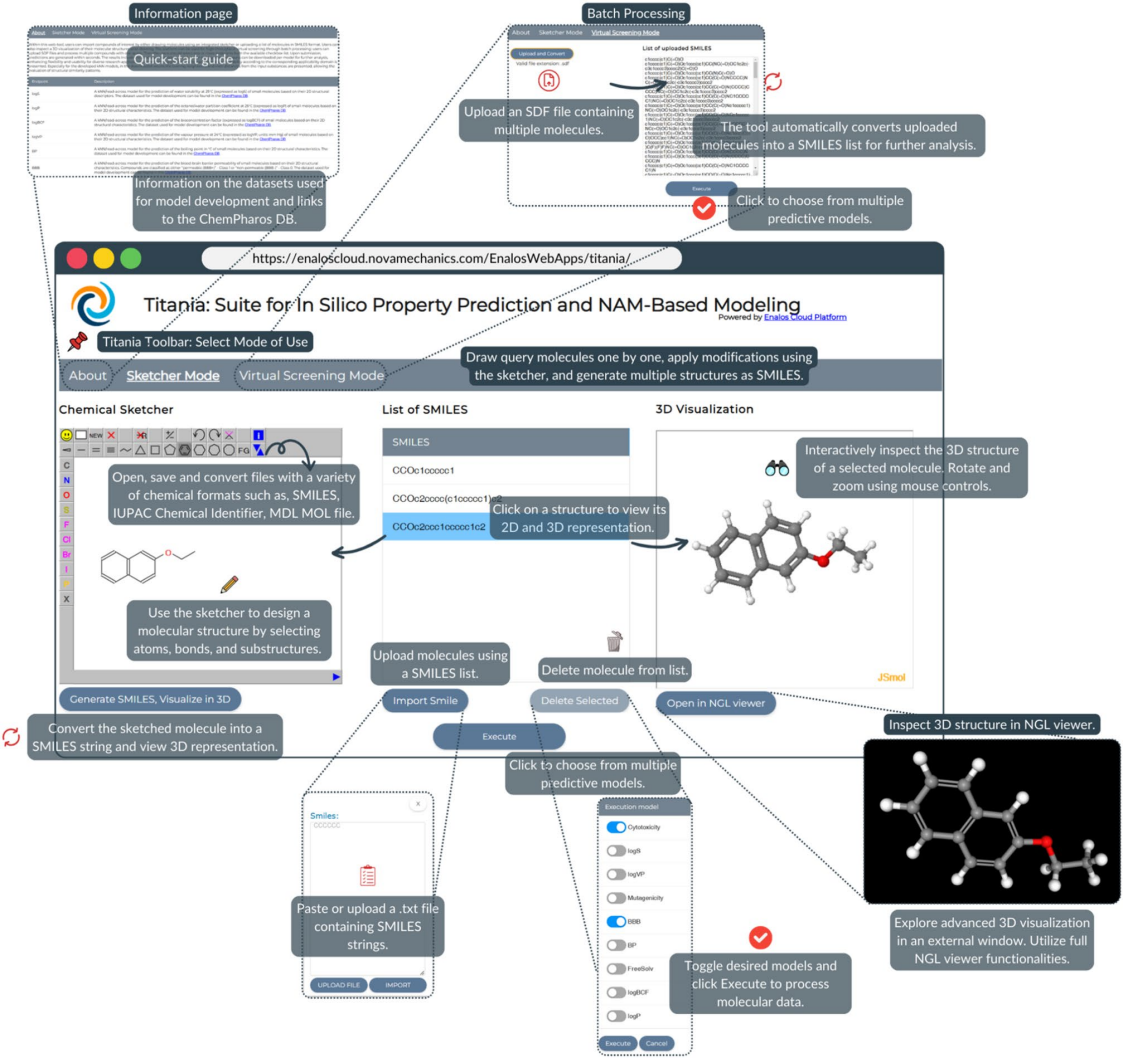
Titania GUI overview. The users can input query compounds by drawing them in the chemical sketcher, uploading them in SMILES format, or as SDF files. 3D visualization options are also available: The users can interactively inspect the molecules’ 3D structure either from the 3D visualization window, or through NGL viewer. Then, they can select the endpoints they want to predict from the list of available models. Links of each dataset used for model development to the ChemPharos DB are also available

The developed and validated models, as described earlier, have been disseminated and made accessible via the intuitive and user-friendly web application Titania (https://enaloscloud.novamechanics.com/EnalosWebApps/titania/), hosted in the Enalos Cloud Platform, thereby facilitating use of the models in drug discovery and materials risk assessment. The graphical user interface (GUI depicted in Fig. 3) allows users to input compounds of interest by either drawing molecules using an integrated sketcher or uploading a single molecule or list of molecules in SMILES format [65]. If the SMILES representation is unknown, users can draw the molecular structure in the chemical sketcher and easily convert it to SMILES. This makes it easy to generate multiple structures by applying various modifications within the sketcher and then converting them to SMILES. The resulting SMILES strings can be used for batch predictions, allowing users to visualize modifications and obtain multiple predictions at once. Users can also inspect a 3D visualization of their molecular structures (without performing energy minimization) post-drawing either in the 3D visualization window or in NGL viewer. The NGL viewer functionality apart from real-time 3D visualization offers multiple atom representation styles, annotation, etc. The Titania platform can be used for high-throughput virtual screening through batch processing: users can upload Spatial Data File (SDF) and process multiple compounds with one query. Then, the users should select one or more endpoints to model from the available checkbox list.

**Fig. 4:**
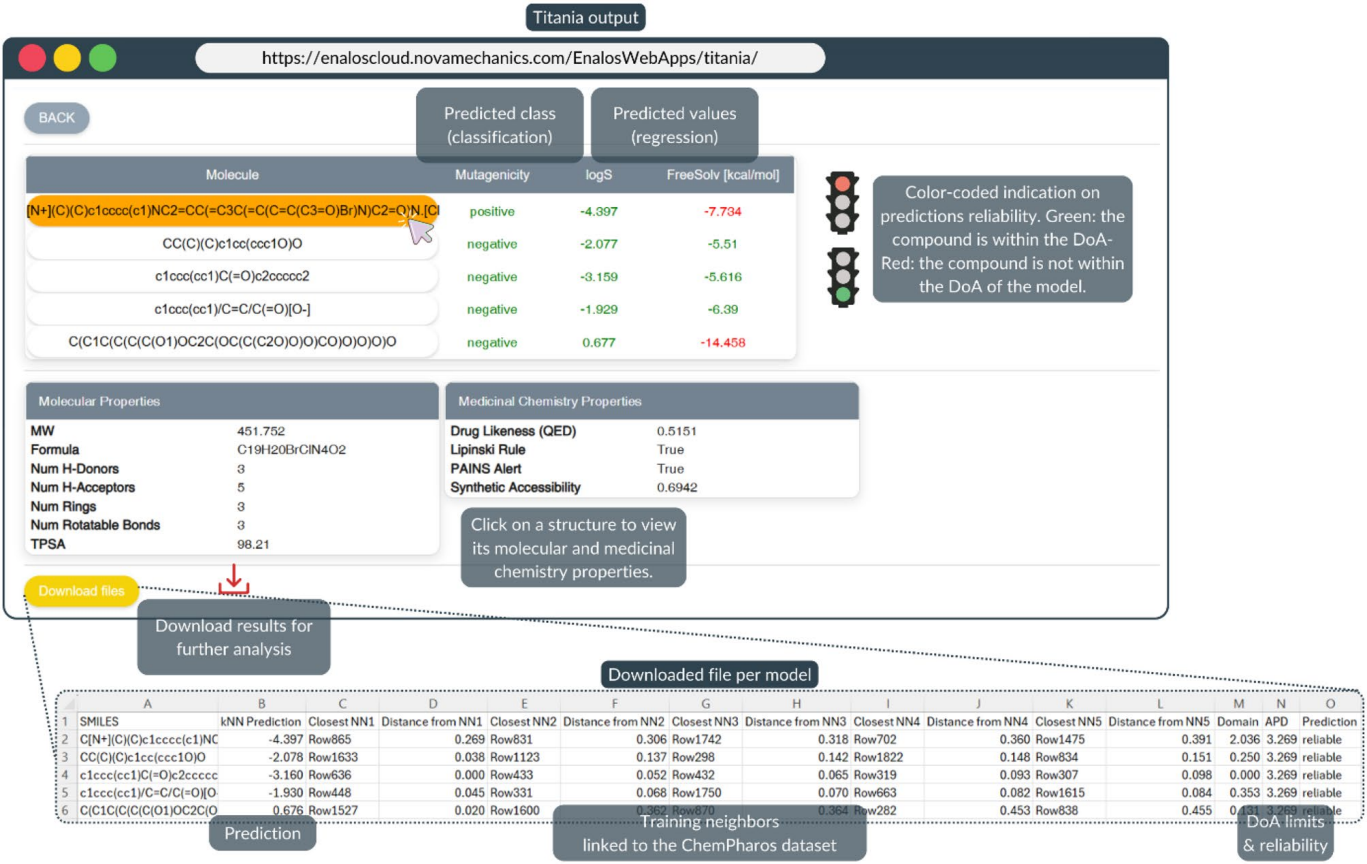
Titania output overview. For each input molecule, predictions for all the selected endpoints are presented. The results include a visual indication of the reliability, where green color indicates the compound is within the DoA of the respective model, and red color indicates that it is not within the DoA of the model. If the molecular and/or the medicinal properties are selected in the input, by clicking on each one of the structures of the output table, these properties are presented

Upon submission, predictions are generated within seconds. The results include the predicted property value(s) and/or predicted class (e.g., permeable or non-permeable) and a color-coded indication of their reliability based on the models’ DoA, whereby green text indicates a reliable prediction, while red text indicates an unreliable one (Figs. 3 and 4). All results can be downloaded per model for further analysis, enhancing flexibility and usability for diverse research applications. In addition to the predictions from each model, their reliability assessment according to the corresponding DoA is presented in the downloaded file. For the developed kNN models, the downloadable results also include the training neighbors and their distances from the input substances, allowing evaluation of the structural similarity patterns. Users can also directly search the k-nearest training neighbors within a dataset used for model development by downloading it from the ChemPharos DB. Specific links to the datasets are available via the “About” tab. For each dataset, apart from the calculated Mold2 descriptors information on the compounds IUPAC name, CID, CAS number, InChIKey, molecular formula and weight, and specific links to the PubChem database are also presented (when available).

### Analysis of alternative web-based tools

Several well-established ADMET prediction tools, including SwissADME [23], ADMETlab (2.0 and 3.0) [25, 26], pkCSM [66], admetSAR (1.0, 2.0 and 3.0) [24, 67, 68], and ADMET-AI [69], have been widely used in drug discovery to estimate pharmacokinetic properties, metabolism, and toxicity. While these platforms offer broad endpoint coverage and predictive capabilities, they often lack model transparency and regulatory compliance—two key areas where Titania stands out. SwissADME specializes in assisting researchers to predict physicochemical properties and drug-likeness of compounds, particularly with consensus logP predictions. ADMETlab (2.0 and 3.0) and admetSAR (1.0, 2.0, and 3.0) provide some of the most comprehensive ADMET endpoint predictions, covering a broad spectrum of absorption, distribution, metabolism, excretion, and toxicity properties. ADMETlab 3.0 has expanded its interpretability features, by incorporating uncertainty estimation scores for prediction results, while admetSAR 3.0 includes the widest range of endpoints, including 119 endpoints. However, these web tools focus on maximizing the endpoint coverage rather than ensuring model explainability and regulatory validation. Titania fills this gap by integrating QMRF compliance, applicability domain estimation for all models and kNN analysis. pkCSM utilizes graph-based signatures for ADMET predictions, often outperforming earlier tools in predictive efficiency. ADMET-AI distinguishes itself with high computational efficiency, allowing for rapid large-scale predictions both online and through local deployment. ADMET-AI provides comparative insights by referencing DrugBank molecules, but it lacks built-in applicability domain validation and mechanistic interpretation.

While existing ADMET tools emphasize speed, endpoint coverage, or computational efficiency, Titania excels in QSAR model transparency, providing QMRF documentation for all models, model interpretability by retrieving the nearest neighbors which contributed to the final prediction of the query compound, and applicability domain assessment. These features allow stakeholders to understand the reasoning behind each property prediction. Moreover, Titania enables researchers to explore chemical modifications dynamically through its sketch section and visualize their molecules in 3D. In Titania’s GUI, molecules can be drawn, modified, and instantly re-evaluated for multiple ADMET properties, allowing multiple modifications on the same scaffold and generation of multiple predictions at once. Additionally, all datasets used for model training and validation are available in the ChemPharos database, ensuring data accessibility and results reproducibility. By providing QSPR/QSTR-ready datasets in standardized formats, along with publicly available models through the Titania web tool, we adhere to the FAIR principles, ensuring that our data and models are Findable, Accessible, Interoperable, and Reusable. This commitment facilitates transparency, reproducibility, and broader adoption of our computational approaches by the scientific community.

However, as with any computational model, Titania has its limitations. While applicability domain estimation enhances reliability, the accuracy of predictions is strongly dependent on the training data. Models may struggle with out-of-distribution compounds, and additional validation with external datasets will be necessary for ongoing refinements. To further enhance its capabilities, Titania will continue to expand its endpoint coverage and retrain models as more high-quality ADMET datasets become available. Despite these challenges, we believe that Titania excels by combining interpretability, usability, and data accessibility, ensuring that the users can perform large-scale ADMET predictions while maintaining a high level of model transparency and regulatory alignment.

### Case studies

We consider three case studies, which illustrate how models are used in the Titania web tool. In these examples, we demonstrate the practical application and effectiveness of the predictive models integrated into the Enalos Cloud Platform. These case studies illustrate the ability of our models to accurately predict molecular properties that are crucial not only in drug discovery but also in material design, such as targeting ligands for material surface modification or optimizing molecular structures for enhanced functionality in advanced materials.

#### Screening of known drugs and tool comparison

To further validate Titania, we conducted a comparative analysis of predicted properties against two widely used ADMET prediction tools, SwissADME [23] and ADMETlab 3.0 [26]. A set of ten approved drugs with diverse chemical properties was selected. In most compounds, Titania’s logP and logS predictions are in agreement with those from other tools, demonstrating consistency in basic physicochemical property estimation. For example, Idoxuridine is a pyrimidine analog antiviral used for the treatment of viral eye infections, including herpes simplex keratitis [70]. Using Titania, logP value was predicted −0.19, which was close to SwissADME and ADMETlab 3.0 values of − 0.20 and − 0.47, respectively. LogS value was predicted − 2.36 using Titania, and − 1.56 and − 2.15 using the other two ADMET tools. Similarly, Norethynodrel has been used for the treatment of functional uterine bleeding and endometriosis [71]. All Titania’s property predictions were in reasonable agreement with those from other tools.

**Fig. 5:**
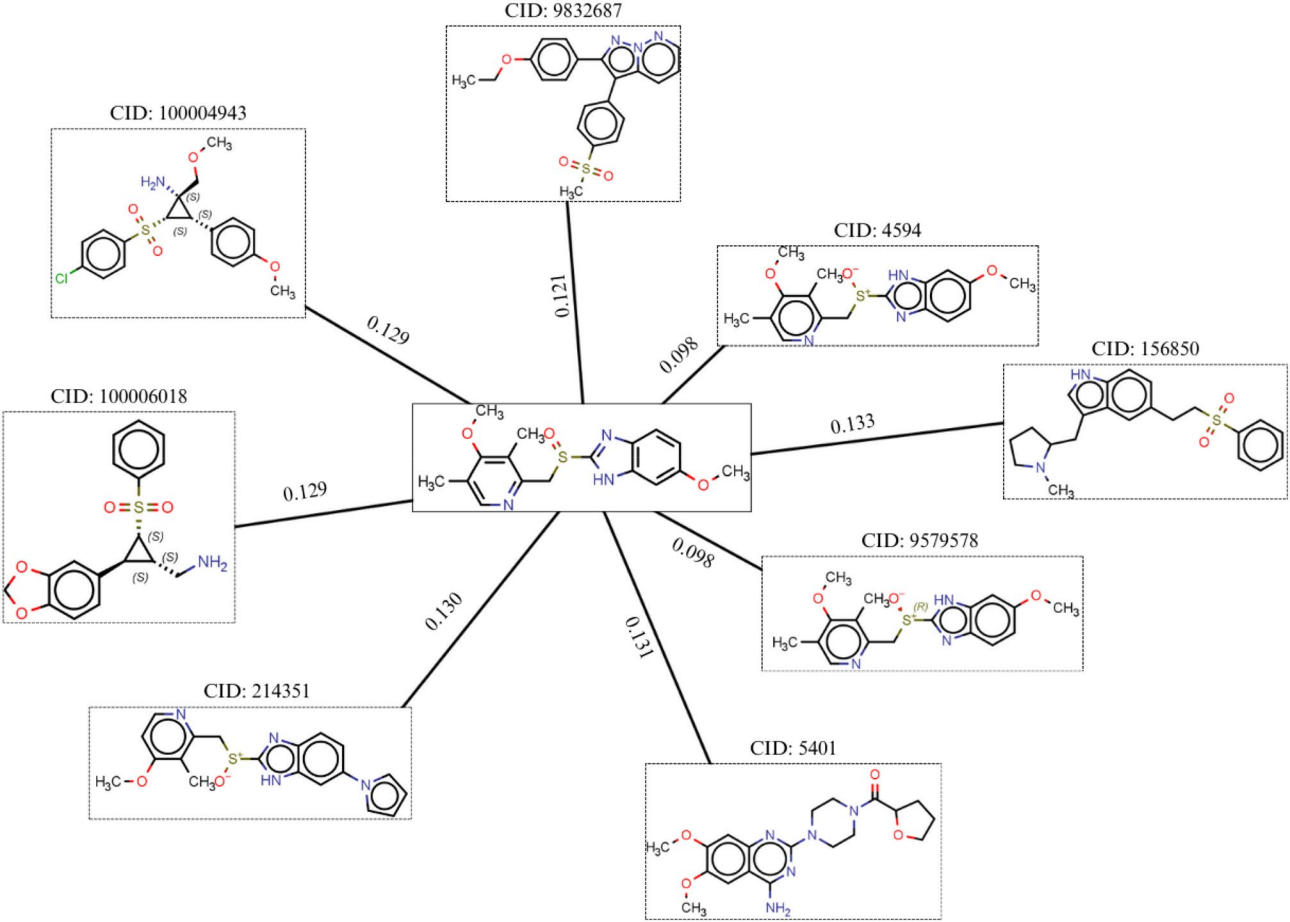
Network of omeprazole with Euclidean distanced from its eight nearest neighbors

In BBB classification, Titania’s predictions aligned with at least one of the two other tools in nine out of ten cases. Sulfaphenazole was predicted as non-permeable (BBB −) in both Titania and SwissADME but as permeable (BBB +) in ADMETlab 3.0, while dibucaine was predicted as permeable only in SwissADME. Notably, omeprazole was predicted as BBB permeable by Titania but as non-permeable by SwissADME and ADMETlab 3.0. Omeprazole is a proton pump inhibitor widely used for the treatment of gastroesophageal reflux disease [72, 73]. Experimental evidence suggests that omeprazole has limited blood–brain barrier penetration. However, there are studies that have reported its ability to penetrate the blood–brain barrier at low concentrations [74–76]. To further investigate Titania’s prediction, we examined its nearest neighbors in chemical space. As shown in Fig. 5, the kNN model identified eight training compounds which contributed to omeprazole’s prediction. Among these, five compounds were permeable, including two with high structural similarity and small Euclidean distances to omeprazole—one of which was permeable and the other non-permeable. The two closest neighbors share the same scaffold but differ in stereochemistry, showing that permeability might favor only one enantiomer [77]. The predominance of permeable neighbors in the kNN space supported Titania’s classification of omeprazole as BBB permeable. However, it should be noted that differences between ADMET tools may arise due to differences in model training data, feature selection, or decision thresholds, especially for classification models. This example highlights an advantage of Titania over other tools, where users can examine the nearest neighbors’ contribution to a prediction. By providing the nearest neighbors, users can have a deeper insight into the model’s decision and assess the reliability of predictions (Table 8).

**Table 8:** Property prediction of known drugs using Titania, SwissADME, and ADMETlab 3.0

Name	Titania			SwissADME			ADMETlab 3.0		
	logP	logS	BBB	logP	logS	BBB	logP	logS	BBB
Sulfathiazole	− 0.11	− 2.23	BBB −	0.81	− 1.77	BBB −	0.15	− 2.63	BBB −
Dibucaine	3.78	− 3.99	BBB −	3.63	− 4.31	BBB +	4.38	− 3.96	BBB −
Idoxuridine	− 0.19	− 2.36	BBB −	− 0.2	− 1.56	BBB −	− 0.47	− 2.15	BBB −
Captopril	0.37	− 2.11	BBB −	0.62	− 1.14	BBB −	0.51	− 0.98	BBB −
Azaguanine-8	− 1.01	− 0.93	BBB −	− 0.73	− 0.94	BBB −	− 0.65	− 3.51	BBB −
Sulfaphenazole	1.60	− 3.25	BBB −	1.62	− 3.05	BBB −	1.15	− 2.76	BBB +
(D) Panthenol	− 1.53	− 0.01	BBB −	− 0.27	− 0.08	BBB −	− 0.09	0.03	BBB −
Sulfadiazine	− 0.24	− 1.91	BBB −	0.4	− 1.66	BBB −	− 0.07	− 3.22	BBB −
Norethynodrel	3.22	− 4.65	BBB +	3.35	− 3.02	BBB +	3.19	− 3.82	BBB +
Omeprazole	2.09	− 3.50	BBB +	2.31	− 3.52	BBB −	1.54	− 2.89	BBB −

#### Screening for PFAS alternatives

Per-and poly-fluoroalkyl substances (PFAS) are synthetic compounds containing at least one fully fluorinated methyl or methylene carbon atom [78]. They have been widely used in textiles, food packaging, surfactants, etc. since the mid-twentieth century [79]. Based on the refined OECD criteria, millions of PFAS-related compounds are currently cataloged in PubChem, classified by chain length and functional groups, influencing their properties and fate. Long-chain PFAS, like perfluorooctane sulfonic acid (PFOS), contain more carbons (8 in this case), while short-chain variants, like perfluorobutanoic acid (PFBA), have fewer (4 in this case) [80].

The persistence and toxicity of PFAS molecules have led to environmental and health concerns, with PFAS detected in water, air, and soil. As the toxicological impact of PFAS compounds on different ecosystems and human health becomes increasingly documented, safety regulators and industry are taking collective actions to reduce their usage in products. Therefore, a preliminary evaluation of potential PFAS substitutes is crucial to gain insight into the environmental impact of alternative compounds before incorporating them into products. By obtaining and assessing the predictions of candidate compounds’ critical properties, such as solubility and mutagenicity, the potential risks posed by untested chemicals can be mitigated prior to their widespread deployment. Such sustainable practices should be encouraged by regulators, to guide research toward assessment of the suitability and safety of alternatives prior to usage thereby preventing the so-called regrettable substitutions in which a chemical with an unknown or unforeseen hazard is used to replace a chemical identified as problematic [81].

Due to the increased attention PFAS have received from environmental regulators, we selected this class of chemicals as a case study to calculate the properties available in Titania. Experimental determination of the physicochemical properties of the vast number of PFAS currently listed in chemical repositories is unfeasible, thus computational tools such as Titania are more practical. Firstly, a query compound was chosen (PubChem CID: 3235567 [82]) after the screening of various bioassays of PFAS-protein interactions extracted from the PubChem BioAssay database. The bioassays contained activity data of small molecules against biological targets, namely the retinoic acid-related orphan receptor (ROR) gamma, the G9a histone methyltransferase, the Human tyrosyl-DNA phosphodiesterase 1 (hTDP1) gene, and P450 cytochromes (CYP2C9, CYP3A4, and CYP2D6) [83]. We searched in the literature for PFAS that do not interact with the above proteins. The query compound was selected since it was classified as ‘inactive’ against all of the mentioned targets, thus we searched on PubChem for similar PFAS compounds that might have more favorable critical properties and are potentially safer for the environment. By tuning the Tanimoto similarity coefficient to 99%, a total of 3 similar PFAS compounds were identified, whose chemical structures are displayed in Table 9 along with their calculated properties. According to the calculated DoA of the models, all predictions are reliable, except for the predictions derived from the Free-Solv model. This is likely attributed to the inherent complexity of PFAS structures and the difference of the identified compounds from the training data available for the Free-Solv model. For the calculated properties, we observe that similar compounds exhibit properties closely aligned with the query compound. The query compound is predicted cytotoxic, while two of the three similar compounds are predicted to be non-cytotoxic; therefore, these compounds might be preferable for further examination.

**Table 9:** Predicted properties of the query compound and similar PFAS compounds

Compound	logP	logS	logBCF	logVP	BP	Mutagenicity	Cytotoxicity
CID: 3235567	3.6	− 4.8	1.66	− 7.2	353.1	Negative	Active
CID: 3234905	4.7	− 6.0	1.60	− 6.3	373.5	Negative	Inactive
CID: 3233189	4.9	− 5.3	1.60	− 6.3	374.6	Negative	Inactive
CID: 3233569	3.9	− 4.5	1.70	− 7.4	352.1	Negative	Active

#### Inhibitors of potential therapeutic targets

A rapid and efficient assessment of the potential toxicity of small molecules represents a field of particular interest in drug discovery since the early evaluation of the toxicity profile of a lead compound is of great importance for its development. Similarly, lipophilicity and aqueous solubility are critical properties of a drug that influence whether it will reach its intended target in the body. Compounds with poor solubility are unable to achieve effective targeting and, therefore, pose a higher risk of attrition and increase overall cost during development.

We selected one potential therapeutic target and inhibitors of this target to calculate their properties. The Endoplasmic Reticulum Aminopeptidase 1 (ERAP1) protein, well known for its aminopeptidase function as a “molecular ruler”, is a crucial enzyme shaping the major histocompatibility complex I (MHC I) immunopeptidome. ERAP1 has been genetically associated with autoinflammatory and autoimmune diseases, such as Ankylosing Spondylitis, Inflammatory bowel disease, psoriasis, and cancer conditions [84]. A selective inhibitor of ERAP1 [85] was selected and used in this case study for prediction of logP, logS, cytotoxicity, mutagenicity, and BBB. We focused on these properties as we consider them important for drug discovery.

We searched for similar compounds to the inhibitor based on Tanimoto similarity to evaluate their properties. Three compounds were identified with similarity of 99% to the query compound. The results of the similarity search are shown in Table 10. From the predicted properties of the similar compounds, we observed that all three were predicted to be cytotoxic. Therefore, it might be beneficial to explore compounds with lower similarity scores, as they might present distinct chemical properties and potentially offer safer alternatives. For similarity of 99% to the query compound, all similar compounds have the same scaffold and they differ only in the phenyl group. Searching for compounds with lower similarity, i.e., 90% to the query compound, might lead to a compound with desired properties.

**Table 10:** Predicted properties of the query compound and similar compounds

Compound	logP	logS	BBB	Mutagenicity	Cytotoxicity
CID: 72193911	− 1.11	− 5.25	Permeable	Negative	Active
CID: 44445781	− 1.45	− 5.36	Non-permeable	Negative	Active
CID: 44445782	− 1.25	− 5.35	Permeable	Negative	Active
CID: 44445783	− 1.22	− 5.39	Permeable	Negative	Active

## Conclusion

This study highlights the critical role of QSPR/QSTR models in predicting molecular properties essential for drug discovery and material design. These NAMs based on computational approaches enable early assessment of chemical endpoints, minimizing experimental costs and risks associated with undesired properties, such as toxicity. We successfully developed in silico models for the prediction of logP, logS, Free-Solv, logVP, BP, cytotoxicity, mutagenicity, BBB, and logBCF. All analysis steps, including data pre-processing, modeling, and validation, are performed on KNIME and the Isalos Analytics platform. The models were developed using the k-nearest neighbors and random forest algorithms and were validated according to the OECD guidelines through external validation and Y-randomization tests. Additionally, the DoA of the models was determined to ensure the reliability of the generated predictions for new compounds that were not involved in model training. Furthermore, a QMRF document accompanies each model, facilitating transparent reporting to the scientific community. Data FAIRness is ensured with data sharing through the ChemPharos database. The developed models are embedded in the Enalos Cloud Platform and are freely available for use by end-users in drug discovery and material design projects. The Titania web tool (https://enaloscloud.novamechanics.com/EnalosWebApps/titania) enables virtual screening of compounds of interest using the validated models, offering an initial in silico testing approach. Titania, and the underpinning Enalos Cloud Platform, aims to become a valuable tool in drug discovery and material research, supporting the reduction of in vitro experiments.

## Supplementary Information

Below is the link to the electronic supplementary material.

Supplementary file1 (ZIP 6052 KB)

Supplementary file2 (DOCX 44 KB)

## Data Availability

chemPharos datasets: https://db.chempharos.eu/datasets/Datasets.zul.
