# Supplementary material for: Titania: an integrated tool for in silico molecular property prediction and NAM-based modeling
Source: Mol Divers. 2025 Apr 23;29(4):3555–73. doi: 10.1007/s11030-025-11196-5 (PMC12245999; doi:10.1007/s11030-025-11196-5)
Supplement: Supplementary file 1 — Supplementary file1 (ZIP 6052 KB) [file 11030_2025_11196_MOESM1_ESM.zip › QMRFs/Cytotoxicity.docx]

# Cytotoxicity–(Q)SAR model reporting format (QMRF) v.2.1

|  | **Element** | **Explanation** |
| --- | --- | --- |
| **1.** | **QSAR identifier** |  |
| 1.1. | QSAR identifier (title) | Read-across model for the correlation of compounds’ structural properties to cytotoxicity to NIH/3T3 cells. |
| 1.2 | Other related models | MouseTox model: <http://www.enaloscloud.novamechanics.com/EnalosWebApps/MouseTox/>  Relevant publication: Varsou, D. D., Melagraki, G., Sarimveis, H., & Afantitis, A. (2017). MouseTox: An online toxicity assessment tool for small molecules through Enalos Cloud platform. Food and Chemical Toxicology, 110, 83-93. https://doi.org/10.1016/j.fct.2017.09.058 |
| 1.3. | Software coding the model | KNIME Analytics Platform v5.1.2  Isalos Analytics Platform  Link to web service:  <https://enaloscloud.novamechanics.com/EnalosWebApps/titania/> |
| **2.** | **General information** |  |
| 2.0 | Abstract | Within this web-tool, users can upload one or several compounds of interest and acquire a prediction of their cytotoxicity to NIH/3T3 cells. The platform provides three different options for inserting the required input to the model: 1. The user can draw the chemical structure of interest using the drawing tool, 2. The user can enter the SMILES notation of one or several compounds separated by newlines and, 3. The user can select and import an SDF file with several compounds. |
| 2.1. | Date of QMRF | 21 October 2024 |
| 2.2. | QMRF author(s) and contact details | Nikoletta-Maria Koutroumpa (koutroumpa@novamechanics.com)  Maria Antoniou (antoniou@novamechanics.com)  Dimitra-Danai Varsou (varsou@novamechanics.com)  Andreas Tsoumanis (tsoumanis@novamechanics.com)  Georgia Melagraki (georgiamelagraki@gmail.com)  Antreas Afantitis (afantitis@novamechanics.com) |
| 2.3. | Date of QMRF update(s) | NA |
| 2.4. | QMRF update(s) | NA |
| 2.5. | Model developer(s) and contact details | Nikoletta-Maria Koutroumpa (koutroumpa@novamechanics.com)  Maria Antoniou (antoniou@novamechanics.com)  Dimitra-Danai Varsou (varsou@novamechanics.com)  Andreas Tsoumanis (tsoumanis@novamechanics.com)  Georgia Melagraki (georgiamelagraki@gmail.com)  Antreas Afantitis (afantitis@novamechanics.com) |
| 2.6. | Date of model development and/or publication | 20 April 2024 |
| 2.7. | Reference(s) to main scientific papers and/or software package | Pending publication |
| 2.8. | Availability of information about the model | The model is proprietary: the source code is confidential; however, the description of the modelling workflow is presented in the original research article, training and validation sets are available as supplementary information of the original research article and the model is implemented as a public web service. |
| 2.9. | Availability of another QMRF for exactly the same model | NA |
| **3** | **Defining the endpoint - OECD Principle 1: “A DEFINED ENDPOINT"** | **PRINCIPLE 1: “A DEFINED ENDPOINT". ENDPOINT refers to any physicochemical, biological, or environmental property/activity/effect that can be measured and therefore modelled. The intent of PRINCIPLE 1 (a (Q)SAR should be associated with a defined endpoint) is to ensure clarity in the endpoint being predicted by a given model, since a given endpoint could be determined by different experimental protocols and under different experimental conditions. It is therefore important to identify the experimental system and test conditions that is being modelled by the Q)SAR.** |
| 3.1. | Species | NIH/3T3 cells (mouse embryonic fibroblast cell line; ATCC CRL-1658) |
| 3.2. | Endpoint | Cytotoxicity to NIH/3T3 cells |
| 3.3 | Comment on endpoint | Categorical endpoint. See also in PubChem: <https://pubchem.ncbi.nlm.nih.gov/bioassay/651744> |
| 3.4. | Endpoint units | The minimum PUBCHEM_ACTIVITY_SCORE required for a compound to be called a hit (the activity threshold, or AT) was set at -24 (Greater than 3 times the standard deviation of the neutral control wells). See also: <https://pubchem.ncbi.nlm.nih.gov/bioassay/651744#section=Comment> |
| 3.5. | Dependent variable | Cytotoxicity: “Active” - Compounds cytotoxic to NIH/3T3s and “Inactive” - Compounds non-cytotoxic to NIH/3T3s. |
| 3.6. | Experimental protocol | See PubChem record: <https://pubchem.ncbi.nlm.nih.gov/bioassay/651744#section=Protocol> |
| 3.7. | Endpoint data quality and variability | Complete dataset:   \| **Active** \| **Inactive** \| **Active/Inactive** \| \| --- \| --- \| --- \| \| 3109 \| 2307 \| 1.35 \| |
| **4** | **Defining the algorithm - OECD Principle 2 : “AN UNAMBIGUOUS ALGORITHM”** | **PRINCIPLE 2: “AN UNAMBIGUOUS ALGORITHM”. The (Q)SAR estimate of an endpoint is the result of applying an ALGORITHM to a set of structural parameters which describe the chemical structure. The intent of PRINCIPLE 2 (a (Q)SAR should be associated with an unambiguous algorithm) is to ensure transparency in the model algorithm that generates predictions of an endpoint from information on chemical structure and/or physicochemical properties. In this context, algorithm refers to any mathematical equation, decision rule or output approach.** |
| 4.1. | Type of model | Instance based/read-across, *k*-Nearest Neighbours (*k*NN) |
| 4.2. | Explicit algorithm | The *k*NN/read-across model employs the *k*-nearest neighbours approach, an instance-based method that predicts the endpoint of a compound based on its *k* nearest neighbours in the data space. The proximity between compounds is measured using Euclidean distance, which is adjusted slightly for categorical descriptor values using a binary value (0 in the case of same class data points or 1, otherwise). The endpoint prediction, in this case the cytotoxicity class, is the weighted majority vote of the class of the *k* closest neighbours (*k* = 8), with each neighbour’s weighting factor inversely proportional to its distance from the evaluated compound. |
| 4.3. | Descriptors in the model | 1. D007: Number of 08-membered rings 2. D025: Number of Nitrogen 3. D217: Valence vertex connectivity order-5 Index 4. D265: Vertex distance information index 5. D326: Distance+detour path with ring index of order 7 6. D336: Distance+detour path on ring index of order 7 (circuits) 7. D354: Molecular topological multiple path index of order 06 8. D358: Molecular topological multiple path index of order 10 9. D361: Ratio of convention bonds with total path counts 10. D374: Sum of topological distance between the vertices O and O 11. D542: Lowest eigenvalue from Burdex matrix weighteed by van der Walls order-3 12. D588: Hightest eignevalue from Burdex matrix weighteed by polarizabilities order-1 13. D589: Hightest eignevalue from Burdex matrix weighteed by polarizabilities order-2 14. D598: Number of total tertiary C-sp3 15. D607: Number of tertiary C-sp2 16. D679: Number of group ethers (aliphatic) 17. D713: Number of group acceptor atoms for H-bonds (N O F) 18. D721: Number of group CHR2X 19. D732: Number of group =CRX |
| 4.4. | Descriptor selection | Feature selection using the “Best First” (bi-directional) approach and the “CfsSubset” evaluator. |
| 4.5. | Algorithm and descriptor generation | Mold2: Mold2 is a software developed by the National Center for Toxicological Research (NCTR) that calculates a large and diverse set of 777 molecular descriptors encoding two-dimensional chemical structure information. <https://www.fda.gov/science-research/bioinformatics-tools/mold2> |
| 4.6. | Software name and version for descriptor generation | Enalos+ KNIME nodes: “EnalosMold2” node that uses the Mold2.exe executable and an SDF file to calculate molecular descriptors. |
| 4.7. | Chemicals/Descriptors ratio | 3791 chemicals/19 descriptors |
| **5** | **Defining the applicability domain - OECD Principle 3: “A DEFINED DOMAIN OF APPLICABILITY”** | **PRINCIPLE 3: “A DEFINED DOMAIN OF APPLICABILITY”. APPLICABILITY DOMAIN refers to the response and chemical structure space in which the model makes predictions with a given reliability. Ideally the applicability domain should express the structural, physicochemical and response space of the model. The CHEMICAL STRUCTURE (x variable) space can be expressed by information on physicochemical properties and/or structural fragments. The RESPONSE (y variable) can be any physicochemical, biological or environmental effect that is being predicted. According to PRINCIPLE 3 a (Q)SAR should be associated with a defined domain of applicability. Section 5 can be repeated (e.g., 5.a, 5.b, 5.c, etc) as many times as necessary if more than one method has been used to assess the applicability domain.** |
| 5.1. | Description of the applicability domain of the model | The applicability domain is defined by fixed boundaries, the APD threshold (§5.2), calculated by considering Euclidean distances between all molecules in the training set. The distance of a test compound to its nearest neighbour in the training set is compared to the predefined applicability domain threshold. If the distance is beyond this threshold, then the prediction is considered unreliable. |
| 5.2. | Method used to assess the applicability domain | The distance of a test molecule to its nearest neighbour in the training set in compared to the pre-defined APD threshold, APD=<d>+stdev*z. First, the average Euclidean distances between all pairs of training data is calculated and then the set of distances that were lower than the average is formulated. The <d> and stdev values are finally determined as the average and standard deviation of all distances included in the remaining set. z is an empirical parameter with a value of 0.5. |
| 5.3. | Software name and version for applicability domain assessment | Isalos Analytics Platform: “Domain – APD” function |
| 5.4. | Limits of applicability | APD threshold = 4.972. |
| **6** | **Defining goodness-of-fit and robustness (internal validation) – OECD Principle 4: “APPROPRIATE MEASURES OF GOODNESS-OF-FIT, ROBUSTENESS AND PREDICTIVITY”** | **PRINCIPLE 4: “APPROPRIATE MEASURES OF GOODNESS-OF-FIT, ROBUSTENESS AND PREDICTIVITY”. PRINCIPLE 4 expresses the need to perform validation to establish the performance of the model. GOODNESS-OF-FIT and ROBUSTNESS refer to the internal model performance.** |
| 6.1. | Availability of the training set | Available at the ChemPharos database: <https://db.chempharos.eu/datasets/Datasets.zul?datasetID=ds5> |
| 6.2. | Available information for the training set | a) Chemical names (common names and/or IUPAC names); b) CAS numbers; c) SMILES; d) InChI codes; e) Structural formula; f) MW. |
| 6.3. | Data for each descriptor variable for the training set | Available at the ChemPharos database: <https://db.chempharos.eu/datasets/Datasets.zul?datasetID=ds5> |
| 6.4. | Data for the dependent variable for the training set | Available at the ChemPharos database: <https://db.chempharos.eu/datasets/Datasets.zul?datasetID=ds5> |
| 6.5. | Other information about the training set | Random splitting (stratified based on the chemicals’ class) was used for partitioning: 3791 out of 5416 molecules were included in the training set for model development. From the remaining 1625 molecules, 1462 were randomly selected as the validation set (not participating in the model development) to test the generated model and adjust its parameters. |
| 6.6. | Pre-processing of data before modelling | Removal of columns that contain the same values at a percentage equal or higher than 80%, Low variance filter (cutoff limit of 10% to filter out columns), Gaussian normalization of descriptors (z-score). Variable selection was performed according to §4.4. |
| 6.7. | Statistics for goodness-of-fit | Training set:   \| **Metric** \| **Value** \| \| --- \| --- \| \| ACC \| 0.913 \| \| SEN \| 0.943 \| \| SPE \| 0.873 \| \| MCC \| 0.822 \|   Validation set:   \| **Metric** \| **Value** \| \| --- \| --- \| \| ACC \| 0.820 \| \| SEN \| 0.857 \| \| SPE \| 0.770 \| \| MCC \| 0.630 \| |
| 6.8. | Robustness - Statistics obtained by leave-one-out cross-validation | \| **Metric** \| **Value** \| \| --- \| --- \| \| ACC \| 0.801 \| \| SEN \| 0.852 \| \| SPE \| 0.731 \| \| MCC \| 0.590 \| |
| 6.9. | Robustness - Statistics obtained by leave-many-out cross-validation | Robustness – Statistics obtained by 5-fold cross-validation (random splitting of data).   \| **Metric** \| **Value** \| \| --- \| --- \| \| ACC \| 0.795 \| \| SEN \| 0.846 \| \| SPE \| 0.725 \| \| MCC \| 0.577 \| |
| 6.10. | Robustness - Statistics obtained by Y-scrambling | \| **Iteration** \| **ACC** \| **SEN** \| **SPE** \| **MCC** \| \| --- \| --- \| --- \| --- \| --- \| \| 1 \| 0.546 \| 0.656 \| 0.398 \| 0.055 \| \| 2 \| 0.543 \| 0.651 \| 0.398 \| 0.050 \| \| 3 \| 0.565 \| 0.682 \| 0.408 \| 0.092 \| \| 4 \| 0.508 \| 0.640 \| 0.329 \| -0.032 \| \| 5 \| 0.536 \| 0.638 \| 0.400 \| 0.038 \| |
| 6.11. | Robustness - Statistics obtained by bootstrap | NA |
| 6.12. | Robustness - Statistics obtained by other methods | NA |
| **7** | **Defining predictivity (external validation) – OECD Principle 4: “APPROPRIATE MEASURES OF GOODNESS-OF-FIT, ROBUSTENESS AND PREDICTIVITY”** | **PRINCIPLE 4: “APPROPRIATE MEASURES OF GOODNESS-OF-FIT, ROBUSTENESS AND PREDICTIVITY”. PRINCIPLE 4 expresses the need to perform validation to establish the performance of the model. PREDICTIVITY refers to the external model validation. Section 7 can be repeated (e.g., 7.a, 7.b, 7.c, etc) as many times as necessary if more validation studies need to be reported in the QMRF.** |
| 7.1. | Availability of the external validation set | Available at the ChemPharos database: <https://db.chempharos.eu/datasets/Datasets.zul?datasetID=ds5> |
| 7.2. | Available information for the external validation set | a) Chemical names (common names and/or IUPAC names); b) CAS numbers; c) SMILES; d) InChI codes; e) Structural formula; f) MW. |
| 7.3. | Data for each descriptor variable for the external validation set | Available at the ChemPharos database: <https://db.chempharos.eu/datasets/Datasets.zul?datasetID=ds5> |
| 7.4. | Data for the dependent variable for the external validation set | Available at the ChemPharos database: <https://db.chempharos.eu/datasets/Datasets.zul?datasetID=ds5> |
| 7.5. | Other information about the external validation set | 163 molecules were included in the external validation (blind) set, which was not involved in model development, but it was rather used solely for validating purposes. |
| 7.6. | Experimental design of test set | Randomly selected from the first partitioning of data (1625 molecules). See also §6.5. |
| 7.7. | Predictivity - Statistics obtained by external validation | \| **Metric** \| **Value** \| \| --- \| --- \| \| ACC \| 0.853 \| \| SEN \| 0.894 \| \| SPE \| 0.797 \| \| MCC \| 0.697 \| |
| 7.8. | Predictivity - Assessment of the external validation set | The external validation set (blind set) is 3% of the initial dataset, 100% of predictions fall within the domain of applicability. |
| 7.9. | Comments on the external validation of the model | Test and validation sets were normalized based on the Gaussian normalization applied on the training set. |
| **8** | **Providing a mechanistic interpretation - OECD Principle 5: “A MECHANISTIC INTERPRETATION, IF POSSIBLE”** | **PRINCIPLE 5: “A MECHANISTIC INTERPRETATION, IF POSSIBLE”. According to PRINCIPLE 5, a (Q)SAR should be associated with a mechanistic interpretation, if possible.** |
| 8.1. | Mechanistic basis of the model | NA |
| 8.2. | A priori or a posteriori mechanistic interpretation | NA |
| 8.3. | Other information about the mechanistic interpretation | NA |
| **9** | **Miscellaneous information** |  |
| 9.1. | Comments | The dataset was retrieved from the PubChem: <https://pubchem.ncbi.nlm.nih.gov/bioassay/651744> |
| 9.2. | Bibliography | 1. Hong, H., Xie, Q., Ge, W., Qian, F., Fang, H., Shi, L., ... & Tong, W. (2008). Mold2, molecular descriptors from 2D structures for chemoinformatics and toxicoinformatics. Journal of chemical information and modeling, 48(7), 1337-1344. <https://doi.org/10.1021/ci800038f> 2. OECD (2014), Guidance Document on the Validation of (Quantitative) Structure-Activity Relationship [(Q)SAR] Models, OECD Series on Testing and Assessment, No. 69, OECD Publishing, Paris, <https://doi.org/10.1787/9789264085442-en>. 3. Varsou, D. D., Melagraki, G., Sarimveis, H., & Afantitis, A. (2017). MouseTox: An online toxicity assessment tool for small molecules through Enalos Cloud platform. Food and Chemical Toxicology, 110, 83-93. <https://doi.org/10.1016/j.fct.2017.09.058> |
| 9.3 | Supporting information | NA |
