# Supplementary material for: Titania: an integrated tool for in silico molecular property prediction and NAM-based modeling
Source: Mol Divers. 2025 Apr 23;29(4):3555–73. doi: 10.1007/s11030-025-11196-5 (PMC12245999; doi:10.1007/s11030-025-11196-5)
Supplement: Supplementary file 2 — Supplementary file2 (DOCX 44 KB) [file 11030_2025_11196_MOESM2_ESM.docx]

Titania: An integrated tool for *in silico* molecular property prediction and NAM-based modeling

Nikoletta-Maria Koutroumpa ^1,2,3^, Maria Antoniou ^1,3,4^, Dimitra-Danai Varsou ^3,5^, Konstantinos D. Papavasileiou ^3,5^, Nikolaos K. Sidiropoulos ^3,5^, Christoforos Kyprianou ^1^, Andreas Tsoumanis ^1,3,5^, Haralambos Sarimveis ^2^, Iseult Lynch ^6^, Georgia Melagraki ^7^, and Antreas Afantitis ^1,3,5^

^1^ NovaMechanics Ltd., Nicosia 1070, Cyprus

^2^ School of Chemical Engineering, National Technical University of Athens, 157 80 Athens, Greece

^3^ Entelos Institute, Larnaca 6059, Cyprus

^4^ Computation-Based Science and Technology Research Center, The Cyprus Institute, Nicosia 2121, Cyprus

^5^ NovaMechanics MIKE., Piraeus, 18545, Greece

^6^ School of Geography, Earth and Environmental Sciences, University of Birmingham, Birmingham, UK

^7^ Division of Physical Sciences & Applications, Hellenic Military Academy, 166 73 Vari, Greece

* Correspondence: [afantitis@novamechanics.com](mailto:afantitis@novamechanics.com)

# Evaluation Metrics

- Correlation of determination ($R^{2}$)

$$R^{2}=1-\frac{\sum_{i=1}^{N} \left( y_{i}-\hat{y_{i}} \right)^{2}}{{\sum_{i=1}^{N} \left( y_{i}-\bar{y} \right)}^{2}} [1]$$

Where $N$, is the number of samples, $y_{i}$ and $\hat{y_{i}}$, are the actual and predicted endpoint values of the $i^{th}$ sample respectively, and $\bar{y}$ and$\hat{y_{i}}$, are the average endpoint values of the experimental and predicted values respectively.

- External explained variance ($Q_{ext}^{2}$)

$$Q_{ext}^{2}=1-\frac{\sum_{i=1}^{N} \left( y_{i}-\hat{y_{i}} \right)^{2}}{\sum_{i=1}^{N} \left( y_{i}-\bar{y}_{train} \right)^{2}} [2]$$

Where $N$, is the number of test samples, $y_{i}$ and $\hat{y_{i}}$, are the actual and predicted endpoint values of the $i^{th}$ test sample respectively. $\bar{y}_{train}$is the average actual activities of the training set samples.

- Mean absolute error (MAE)

$$MAE= \frac{1}{N}\sum_{i=1}^{N} \left| y_{i}-\hat{y_{i}} \right| [3]$$

Where $N$, is the number of samples, $y_{i}$ and $\hat{y_{i}}$, are the actual and predicted endpoint values of the $i^{th}$ sample respectively.

- Mean square error (MSE)

$$MSE= \frac{1}{N}\sum_{i=1}^{N} \left( y_{i}-\hat{y_{i}} \right)^{2} [4]$$

Where $N$, is the number of samples, $y_{i}$ and $\hat{y_{i}}$, are the actual and predicted endpoint values of the $i^{th}$ sample respectively.

- Root mean square error (RMSE)

$$RMSE=\sqrt{\frac{1}{N}\sum_{i=1}^{N} \left( y_{i}-\hat{y_{i}} \right)^{2}} [5]$$

Where $N$, is the number of samples, $y_{i}$ and $\hat{y_{i}}$, are the actual and predicted endpoint values of the $i^{th}$ sample respectively.

- Concordance correlation coefficient (CCC)

$$CCC=\frac{2\sum_{i=1}^{n_{EXT}} \left( Y_{i}-\bar{Y} \right)\left( Y_{i^{'}}-\bar{Y_{i^{'}}} \right)}{{\sum_{i=1}^{n\_EXT} (Y_{i}-\bar{Y})}^{2}+ {\sum_{i=1}^{n\_EXT} (Y_{i^{'}}-\bar{Y_{i^{'}}})}^{2}+{n_{EXT}(Y_{i^{'}-}\bar{Y_{i^{'}}})}^{2}} [6]$$

Where $Y_{i}$ is the experimental value, $\bar{Y}$ is the average of experimental values, $Y_{i^{'}}$ is the predicted value of activity and $\bar{Y}_{i}$ is the average of the predicted value of the activity. EXT is the external prediction set or test set [1], [2].

In addition to the above metrics, the test for an acceptable regression QSAR model was conducted as proposed by Golbraikh and Tropsha [3].

Different metrics were employed for the classification models, since they aim to predict the class of a target NM and not a numerical endpoint. The performance of these models is evaluated based on the number of correct predictions and the number of misclassifications. The statistics used to assess the goodness-of-fit and predictivity of the developed classification models are [4]:

- Sensitivity or Recall (True Positive Rate, TPR)

$$TPR=\frac{TP}{TP+FN} [7]$$

- Specificity (True Negative Rate, TNR)

$$TNR=\frac{TN}{TN+FP} [8]$$

- Accuracy (ACC)

$$ACC=\frac{TP+TN}{TP+TN+FP+FN} [9]$$

- Matthews Correlation Coefficient (MCC)

$$MCC=\frac{TP\times TN-FP\times FN}{\sqrt{(TP+FP)(TP+FN)(TN+FP)(TN+FN)}} [10]$$

# Comparison of model performance

Table S1: Evaluation metrics of machine learning models on validation dataset for regression tasks

| Property | Model | R^2^ | MAE | RMSE | CCC |
| --- | --- | --- | --- | --- | --- |
| logP | RF | 0.853 | 0.491 | 0.694 | 0.911 |
|  | kNN | 0.850 | 0.487 | 0.682 | 0.914 |
|  | SVM | 0.837 | 0.487 | 0.699 | 0.914 |
|  | MLP | 0.819 | 0.529 | 0.767 | 0.909 |
| logS | RF | 0.872 | 0.485 | 0.659 | 0.921 |
|  | kNN | 0.870 | 0.524 | 0.699 | 0.922 |
|  | SVM | 0.872 | 0.479 | 0.659 | 0.921 |
|  | MLP | 0.843 | 0.525 | 0.514 | 0.906 |
| Free-Solv | RF | 0.845 | 0.971 | 1.491 | 0.913 |
|  | kNN | 0.856 | 0.879 | 1.436 | 0.922 |
|  | SVM | 0.835 | 0.891 | 1.536 | 0.905 |
|  | MLP | 0.829 | 1.050 | 1.561 | 0.911 |
| logVP | RF | 0.901 | 0.761 | 1.199 | 0.937 |
|  | kNN | 0.893 | 0.736 | 1.148 | 0.943 |
|  | SVM | 0.891 | 0.711 | 1.141 | 0.934 |
|  | MLP | 0.901 | 0.766 | 1.205 | 0.935 |
| BP | RF | 0.856 | 20.641 | 29.651 | 0.928 |
|  | kNN | 0.869 | 20.174 | 30.872 | 0.929 |
|  | SVM | 0.819 | 23.321 | 36.132 | 0.896 |
|  | MLP | 0.856 | 20.545 | 30.863 | 0.919 |
| logBCF | RF | 0.808 | 0.365 | 0.475 | 0.895 |
|  | kNN | 0.833 | 0.350 | 0.445 | 0.911 |
|  | SVM | 0.801 | 0.378 | 0.489 | 0.893 |
|  | MLP | 0.781 | 0.405 | 0.509 | 0.879 |

Table S2: Evaluation metrics of machine learning models on validation dataset for classification tasks

| Property | Model | SEN | SPE | ACC | MCC |
| --- | --- | --- | --- | --- | --- |
| Cytotoxicity | RF | 0.851 | 0.772 | 0.820 | 0.631 |
|  | kNN | 0.857 | 0.770 | 0.820 | 0.630 |
|  | SVM | 0.859 | 0.731 | 0.815 | 0.618 |
|  | MLP | 0.848 | 0.772 | 0.816 | 0.623 |
| Mutagenicity | RF | 0.792 | 0.811 | 0.802 | 0.602 |
|  | kNN | 0.801 | 0.692 | 0.755 | 0.504 |
|  | SVM | 0.701 | 0.669 | 0.691 | 0.378 |
|  | MLP | 0.831 | 0.690 | 0.767 | 0.529 |
| BBB | RF | 0.926 | 0.763 | 0.866 | 0.701 |
|  | kNN | 0.940 | 0.753 | 0.872 | 0.720 |
|  | SVM | 0.957 | 0.705 | 0.865 | 0.701 |
|  | MLP | 0.920 | 0.777 | 0.868 | 0.712 |

# Descriptor Contributions and Model Overlap

Table S3: Overlap of descriptors with models

| Descriptor | logP | logS | logBCF | logVP | BP | Cytotoxicity | Mutagenicity | BBB | FreeSolv |
| --- | --- | --- | --- | --- | --- | --- | --- | --- | --- |
| D025 |  |  |  | X |  | X |  |  |  |
| D123 |  |  |  |  |  |  | X | X |  |
| D195 |  |  | X |  |  |  |  |  | X |
| D241 |  | X |  |  | X |  |  |  |  |
| D262 |  |  |  | X | X |  |  |  |  |
| D274 |  |  |  | X |  |  |  |  | X |
| D279 |  |  |  |  | X |  |  | X |  |
| D282 | X | X |  |  |  |  |  |  |  |
| D300 |  |  |  |  | X |  | X |  | X |
| D354 |  | X |  | X | X | X | X |  |  |
| D374 |  |  |  |  |  | X |  | X |  |
| D439 |  |  |  | X | X |  |  |  |  |
| D503 | X | X | X |  |  |  |  | X |  |
| D567 | X | X |  | X |  |  |  |  |  |
| D572 |  |  |  |  |  |  | X | X |  |
| D589 |  |  |  |  | X | X |  |  |  |
| D592 |  |  |  |  | X |  |  | X |  |
| D593 |  |  |  | X |  |  |  | X |  |
| D604 | X | X |  |  | X |  | X |  |  |
| D712 | X |  |  |  |  |  |  | X | X |
| D713 |  |  |  |  |  |  |  |  | X |
| D732 |  |  |  |  |  | X |  | X |  |
| D747 |  |  |  | X |  |  |  | X |  |
| D775 | X | X | X |  |  |  |  | X |  |
| D777 | X | X | X |  |  |  |  | X |  |

Where:

Table S4: Description of selected Mold2 molecular descriptors [5].

| Descriptor | Descriptor Name |
| --- | --- |
| D025 | number of Nitrogen |
| D123 | average of molecular weight |
| D195 | maximal valence vertex electrotopological positive variation |
| D241 | average longest path of the molecule |
| D262 | information of bonds index |
| D274 | information content order-5 index |
| D279 | total information content order-4 index |
| D282 | structural information content order-1 index |
| D300 | spanning tree with log value |
| D354 | molecular topological multiple path index of order 06 |
| D374 | sum of topological distance between the vertices O and O |
| D439 | topological structure autocorrelation length-1 weighted by atomic polarizabilities |
| D503 | moran topological structure autocorrelation length-1 weighted by atomic polarizabilities |
| D567 | highest eigenvalue from Burden matrix weighted by masses order-4 |
| D572 | highest eigenvalue from Burden matrix weighted by van der Walls order-1 |
| D589 | highest eigenvalue from Burden matrix weighted by polarizabilities order-2 |
| D592 | highest eigenvalue from Burden matrix weighted by polarizabilities order-5 |
| D593 | highest eigenvalue from Burden matrix weighted by polarizabilities order-6 |
| D604 | number of substituted aromatic C-sp2 |
| D712 | number of group donor atoms for H-bonds (with N and O) |
| D713 | number of group acceptor atoms for H-bonds (N O F) |
| D732 | number of group =CRX |
| D747 | number of group H attached to heteroatom |
| D775 | hydrophilic factor index |
| D777 | molecular regresson coefficients surface LogP index |

# References

[1] P. Gramatica, S. Cassani, P. P. Roy, S. Kovarich, C. W. Yap, and E. Papa, “QSAR Modeling is not ‘Push a Button and Find a Correlation’: A Case Study of Toxicity of (Benzo‐)triazoles on Algae,” *Mol. Inform.*, vol. 31, no. 11–12, pp. 817–835, Dec. 2012, doi: 10.1002/minf.201200075.

[2] S. Shayanfar and A. Shayanfar, “Comparison of various methods for validity evaluation of QSAR models,” *BMC Chem.*, vol. 16, no. 1, p. 63, Aug. 2022, doi: 10.1186/s13065-022-00856-4.

[3] A. Golbraikh and A. Tropsha, “Beware of q2!,” *J. Mol. Graph. Model.*, vol. 20, no. 4, pp. 269–276, Jan. 2002, doi: 10.1016/S1093-3263(01)00123-1.

[4] M. Z. Naser and A. H. Alavi, “Error Metrics and Performance Fitness Indicators for Artificial Intelligence and Machine Learning in Engineering and Sciences,” *Archit. Struct. Constr.*, vol. 3, no. 4, pp. 499–517, Dec. 2023, doi: 10.1007/s44150-021-00015-8.

[5] H. Hong *et al.*, “Mold^2^ , Molecular Descriptors from 2D Structures for Chemoinformatics and Toxicoinformatics,” *J. Chem. Inf. Model.*, vol. 48, no. 7, pp. 1337–1344, Jul. 2008, doi: 10.1021/ci800038f.
